# Da Yuan Yin Regulates Gut Microbiota and Improves Intestinal Injury in Sepsis

**DOI:** 10.1002/fsn3.71456

**Published:** 2026-04-03

**Authors:** Li Wang, Yuan‐Yuan Sha, Guan‐Yu Hu, Yi‐Ming Qian, Jian Guo

**Affiliations:** ^1^ Department of Emergency Medicine, Yueyang Hospital of Integrated Traditional Chinese and Western Medicine Shanghai University of Traditional Chinese Medicine Shanghai China

**Keywords:** Da Yuan Yin, gut microbiota, intestinal injury, sepsis

## Abstract

Sepsis is a common disease, which is a life‐threatening organ dysfunction caused by the host's dysfunctional response to infection. Da Yuan Yin (DYY) is a traditional Chinese medicine that has anti‐inflammatory and purgative effects. Sepsis mouse model was conducted by lipopolysaccharide induction to explore the effects of DYY in vivo. Hematoxylin–eosin staining was performed to observe mouse ileum tissue. ELISA and western blot were carried out to measure the levels of inflammatory factors and tight junction proteins. Moreover, proliferation and apoptosis were measured by immunohistochemistry (Ki67) and TUNEL staining. 16S rRNA sequencing was implemented to predict the effects of DYY on gut microbiota in sepsis. Metabolic function was predicted by PICRUSt2 and experimentally validated by measuring short‐chain fatty acids (SCFAs) and β‐glucuronidase activity. A fecal microbiota transplantation (FMT) experiment was performed to establish causality. In this study, DYY alleviated sepsis‐induced intestinal injury. Additionally, DYY inhibited inflammation (TNF‐α, IL‐1β, and IL‐6), cell apoptosis, and promoted proliferation in sepsis, as well as the promotion of tight junction proteins (claudin‐1, occludin, and ZO‐1). 16S rRNA sequencing revealed that DYY could regulate the alteration in the abundance of gut microbiota in sepsis and promote the growth of bacilli, such as Lactobacillales and Enterobacteriaceae. Functionally, DYY increased protective SCFA levels and suppressed β‐glucuronidase activity. Crucially, FMT from DYY‐treated donors replicated these protective effects in septic recipients, directly demonstrating the mediatory role of gut microbiota. Collectively, DYY can mitigate intestinal injury and modulates gut microbiota in sepsis; the protective role of DYY on sepsis was mediated through the regulation of gut microbiota, which may be a promising therapeutic strategy for sepsis.

## Graphical abstract

Sepsis mouse model was induced by LPS to investigate the effect of Da Yuan Yin (DYY) in vivo. Hematoxylin–eosin staining showed that DYY could reduce intestinal injury caused by sepsis. In addition, DYY can also inhibit inflammation, apoptosis, and promote cell proliferation in sepsis, and promote the expression of tight junction proteins. 16S rRNA sequencing and metabolome sequencing showed that DYY could regulate the abundance of intestinal microbiota in sepsis.

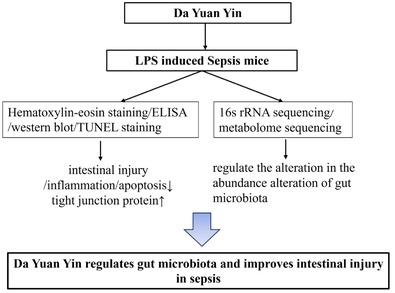

## 1 Introduction

Sepsis is a common disease that is an organ dysfunction induced by the host's dysfunctional response to infection, and in severe cases, sepsis can be life‐threatening (Liu et al. 2022; Heming et al. 2021). Sepsis has a high long‐term incidence worldwide, accompanied by high mortality. Sepsis is a systemic immune disorder or inflammatory response process and is also associated with functional changes of multiple organs in the body (Nedeva 2021). The pathogenesis of sepsis is exceedingly complicated, including inflammatory response imbalance, endoplasmic reticulum stress, coagulation dysfunction, autophagy, neuroendocrine immune network abnormalities, immune dysfunction, and other physiological processes, eventually leading to organ dysfunction (Huang et al. 2019). Currently, treatments for sepsis focus on infection control, hemodynamic management, and host response modulation (Vincent 2022). Sepsis often leads to a variety of complications, such as brain dysfunction, cardiomyopathy, and liver injury (Pan et al. 2022; L'Heureux et al. 2020; Sun et al. 2020). Therefore, it is still the focus to actively explore the pathogenesis and find underlying therapeutic strategies for sepsis.

Sepsis is characterized by a dysregulated host response to infection, and the gut microbiota can modulate homeostasis, including advocating barrier protection and immunity, so microorganisms may play a significant role in the pathogenesis of sepsis, and alterations in the gut microbiota can affect susceptibility to sepsis (Adelman et al. 2020). In the past 30 years, targeting the gut microbiota has been applied to improve the prognosis of critically ill patients with sepsis and has been shown to be effective. A synbiotic preparation ( *Lactobacillus plantarum* and oligofructose) is effective in reducing mortality in neonatal sepsis (Panigrahi et al. 2017). Supplementation of fiber in the diet alters the gut microbiota in septic mice and provides protection for septic mice (Morowitz et al. 2017). Therefore, targeting the gut microbiota is an effective treatment to reduce sepsis mortality, and it is necessary to explore the species of flora in the gut of septic mice.

Traditional Chinese Medicine (TCM) is widely recognized in the treatment of various diseases due to its remarkable therapeutic effects and minimal side effects (Zhou et al. 2017). Da Yuan Yin (DYY) is a traditional Chinese medicine formula consisting of seven herbs: betel nut, Houpu (*Magnolia officinalis*), Caoguo (*Amomum tsaoko*), Zhimu (*Anemarrhena asphodeloides*), *Paeonia lactiflora* , *Scutellaria baicalensis*, and Licorice (*Glycyrrhiza uralensis Fisch*), which has anti‐inflammatory and purgative effects. Researches have been supported that DYY can inhibit inflammation, complement and oxidative stress to attenuate LPS‐induced acute lung injury (ALI), which can be applied as a hopeful therapeutic agent for ALI (Guo et al. 2023; Yang et al. 2023). In addition, DYY has exerted significant effects in the treatment of coronavirus disease 2019 (Ruan et al. 2020). However, the influence of DYY on sepsis is still unknown. Herein, we constructed the sepsis mouse model to explore the effects of DYY in vivo, and the effects of DYY on the intestinal flora species and metabolic pathways of septic mice were investigated by 16S rRNA sequencing and metabolomics sequencing techniques.

## 2 Materials and Methods

### 2.1 High‐Performance Liquid Chromatography–Tandem Mass Spectrometry (HPLC‐MS/MS)

To characterize the chemical constituents of DYY granules, a non‐targeted metabolomics approach was performed using HPLC‐MS/MS. Briefly, 500 μL of DYY extract was centrifuged at 12,000 rpm for 15 min at 4°C, and the supernatant was collected after repeating the centrifugation step. Then, 300 μL of supernatant was diluted with an equal volume of ultrapure water containing 5 ppm L‐2‐chlorophenylalanine, vortexed for 60 s, and filtered through a 0.22‐μm membrane. Quality control (QC) samples were prepared by pooling 10–20 μL of each filtered sample. Chromatographic separation was carried out on a Thermo Vanquish Flex system equipped with an ACQUITY UPLC HSS T3 column (1.8 μm, 2.1 × 100 mm) maintained at 40°C. The mobile phase consisted of 0.1% formic acid in water (A) and 0.1% formic acid in acetonitrile (B) with a gradient elution program: 5% B to 95% B over 0–8.5 min, followed by re‐equilibration to 5% B. The flow rate was 0.4 mL/min, and the injection volume was 2 μL. Mass spectrometric data were acquired on a Thermo Orbitrap Exploris 120 instrument equipped with a HESI ion source. The spray voltages were set at +3.5 kV (positive mode) and −3.0 kV (negative mode). Full‐scan MS data were collected in the *m*/*z* range of 70–1000 with a resolution of 60,000, and data‐dependent acquisition (DDA) mode was applied for MS/MS scans (resolution 15,000, HCD collision energy 30%).

### 2.2 Establishment of Sepsis Mouse Model

6‐week‐old C57BL/6 male mice (18–20 g) were obtained from SiPeiFu Biotechnology (Beijing, China), acclimatized and fed for a week, and randomly divided into Control (Control), Model (SEPSIS), High‐dose DYY (H‐DYY, 35 mg DYY/8.4 mL saline), and Low‐dose DYY (L‐DYY, 30 mg DYY/8.4 mL saline) groups on average (*n* ≥ 6). Each mouse was gavaged with 0.2 mL DYY solution per day (Guo et al. 2023; Yang et al. 2023). After 7 days, except for the control group, mice in the other three groups received an intraperitoneal injection of 5 mg/kg lipopolysaccharide (LPS) to construct the sepsis model. After modeling for 48 h, all mice were anesthetized with isoflurane inhalation and sacrificed by cervical dislocation, and the ileum tissues and serum were collected for subsequent experiments. All animal experiments have been known and approved by the local ethics review committee.

### 2.3 16S rRNA Sequencing

The intestinal contents in each group were collected, and microbial genomic DNA was extracted from the intestinal contents of mice using E.Z.N.A. Soil DNA Kit (Omega Biotek Norcross, GA, USA). The V3 and V4 regions of the 16S rRNA gene were amplified using the ABI GeneAmp 9700 PCR Thermal Cycler (Applied Biosystems, Foster City, CA, USA). TruSeq Nano DNA LT Library Prep Kit (Illumina, CA, USA) was used to prepare the sequencing library, and the MiSeq sequencer was used for paired‐end sequencing. QIIME2 was used for alpha diversity analysis, and the box plot was drawn using the R language. To determine the differences in the composition of intestinal microbes in each group, beta diversity analysis was performed to explore the similarities or differences in community composition between different groups of samples.

### 2.4 Metabolome Sequencing

The intestinal contents were collected, and the microbial genomic DNA was extracted from the intestinal contents of mice using E.Z.N.A. Soil DNA Kit (Omega Biotek Norcross, GA, USA). The V3 and V4 regions of the 16S rRNA gene were amplified and sequenced. Referring to the known microbial genome data, the sequencing results were used to predict the composition of bacterial genes or functional units. PICRUSt2 analysis, PCoA analysis, and species composition and difference analysis of metabolic pathways were performed.

### 2.5 Fecal Microbiota Transplantation (FMT)

To directly investigate whether the protective effects of DYY were mediated by gut microbiota, an FMT experiment was performed as previously described with modifications (Bao et al. 2023). Male C57BL/6 mice (SiPeiFu Biotechnology) were divided into donor and recipient groups. Donor mice were treated with either a high dose of DYY or an equal volume of PBS via daily gavage for 7 days prior to LPS‐induced sepsis modeling. Intestinal contents from donors were collected aseptically 48 h post‐modeling, suspended in sterile PBS, vortexed for 10 s, and centrifuged at 800 g for 3 min. The supernatant was collected as fresh transplant material on the day of administration. Recipient mice were randomly assigned to five groups (*n* ≥ 6): (1) Control: non‐septic mice receiving FMT from healthy donors; (2) SEPSIS: LPS‐induced septic mice without FMT; (3) SEPSIS + SEPSIS − FMT: septic mice receiving FMT from septic donor mice; (4) SEPSIS + PBS − FMT: septic mice receiving FMT from healthy (PBS‐treated) donor mice; (5) SEPSIS + DYY − FMT: septic mice receiving FMT from DYY‐treated septic donor mice. Recipients received 100 μL of the respective fresh fecal suspension via oral gavage 30 min after LPS injection and were sacrificed 48 h later for sample collection. Ileum tissues and serum were collected for further analyses.

### 2.6 Enzyme Linked Immunosorbent Assay (ELISA)

The levels of tumor necrosis factor‐alpha (TNF‐α), interleukin‐1 beta (IL‐1β), and interleukin‐6 (IL‐6) in ileum tissues were measured using the corresponding ELISA kits (Esebio, Shanghai, China). Total 50 μL of diluted samples and standards were supplemented to the wells of the microtiter plate. Later, horseradish peroxidase (HRP)‐labeled antibody (100 μL) was added to each well, and the plates were incubated for 60 min at 37°C. The optical density (OD) value was determined at 450 nm within 15 min. The levels of TNF‐α, IL‐1β, and IL‐6 were evaluated by standard curve.

### 2.7 Hematoxylin–Eosin (HE) Staining

The ileum tissue of mice was fixed in 4% paraformaldehyde solution and dehydrated with ethanol gradient after cleaning. After that, the tissue was transparent with xylene and then embedded in paraffin. The tissue was cut into 4‐μm slices on the slicer, and the maximum cross section of the ileum tissue was taken. Paraffin‐embedded sections were routinely deparaffinized with xylene and ethanol and stained sequentially with hematoxylin and eosin. Subsequently, slices were treated with gradient ethanol and xylene and finally sealed with neutral gum. The staining results were observed under an optical microscope.

### 2.8 Immunohistochemistry (IHC)

Paraffin sections were placed in xylene and gradient ethanol for dewaxing and rehydration, and then the sections were washed and placed in sodium citrate (10 mmol/L) for antigen repair. At room temperature, slices were incubated with 0.3% H_2_O_2_ to quench endogenous peroxidase activity. Then, the slices were blocked with bovine serum albumin (BSA) and incubated with specific primary antibody (anti‐Ki67, ab15580, 1:400, Abcam, Shanghai, China) at 4°C overnight. Then, the slices were incubated with HRP‐labeled goat anti‐rabbit IgG secondary antibody (ab6721, 1:200, Abcam) at 37°C for 1 h. Subsequently, slices were dyed with DAB and hematoxylin, and finally sealed and observed.

### 2.9 Western Blot Assay

The ileum tissues were treated with RIPA to isolate total protein samples. Proteins were separated by 10% sodium dodecyl sulfate polyacrylamide gel electrophoresis and then transferred to a polyvinylidene fluoride (PVDF) membrane. Subsequently, the membrane was sealed in 5% skimmed milk and then incubated with primary antibodies at 4°C for a night, including anti‐Claudin‐1 (ab211737, 1:1000, Abcam), anti‐Occludin (ab216327, 1:1000, Abcam), anti‐ZO‐1 (ab221547, 1:1000, Abcam), and anti‐β‐actin (ab8227, 1:1000, Abcam). Subsequently, the membrane was incubated with the goat anti‐rabbit secondary antibody (1:5000, Abcam) for 1 h at room temperature. At last, the membrane was placed on the plate and added to ECL solution for developing. The image was observed and calculated by Image J software.

### 2.10 Terminal Deoxynucleotidyl Transferase‐dUTP Nick‐End Labeling (TUNEL) Staining

Paraffin sections were rehydrated with xylene and gradient ethanol. The sections were then incubated in proteinase K solution at 37°C for 15 min. After that, the slices were washed with PBS and dried. A total of 50 μL of TUNEL reaction mixture (solution A: solution B = 1: 9) was added to the slides and incubated at 37°C for 1 h. After that, diaminobenzidine and hematoxylin staining were used. After dehydration and transparency, the slices were sealed with neutral gum, and finally observed under the microscope. The cumulative fluorescence value was measured by Image‐pro plus.

### 2.11 Measurement of Short‐Chain Fatty Acids (SCFAs) and β‐Glucuronidase Activity

To functionally validate changes in microbial metabolism, levels of major SCFAs (acetate, propionate, butyrate) were measured in intestinal content samples using corresponding kits. β‐glucuronidase activity in mouse intestinal contents was quantified using a β‐glucuronidase assay kit. Intestinal contents were homogenized in PBS, centrifuged at 10,000× *g* for 10 min at 4°C, and the supernatant was incubated with substrate at 37°C for 30 min. The absorbance was measured for activity calculation.

### 2.12 Quantitative Real‐Time Polymerase Chain Reaction (qRT‐PCR)

Total RNA was extracted from mouse ileum tissues using TRIzol Reagent. RNA quality was quantified via a NanoDrop One spectrophotometer (Thermo Fisher Scientific, Waltham, MA, USA). Complementary DNA synthesis was performed with 1 μg of total RNA and PrimeScript RT Master Mix (Takara Bio, Otsu, Shiga, Japan). Quantitative PCR was executed using SYBR Green Master Mix (Servicebio, Wuhan, China), with thermal cycling parameters: 95°C for 30 s, followed by 40 cycles of 95°C for 10 s, 60°C for 30 s, and a subsequent melt curve analysis (60°C–95°C). Gene expression levels were normalized to glyceraldehyde‐3‐phosphate dehydrogenase (GAPDH) and calculated using the 2^−ΔΔCt^ method. The primer sequences were detailed in Table S1.

### 2.13 Statistical Analysis

For 16S rRNA sequencing, significant abundance differences between multiple groups were detected using the Kruskal–Wallis sum test, and between‐group differences were analyzed using the dunn's test. Finally, linear discriminant analysis (LDA) in Lefse was used to estimate the effect of differential characteristics on the effect of differences (LDA score values) for each group. The corresponding *p*‐values for the differentially abundant taxa identified in the LEfSe analysis were provided in Table S2.

Data were analyzed using GraphPad Prism 8.0 and expressed as mean ± standard deviation. A *t*‐test was performed for comparisons between two groups, and one‐way analysis of variance (ANOVA) and Turkey's test were used for multiple comparisons. *p* < 0.05 were considered statistically significant.

## 3 Results

### 3.1 Chemical Constituents of DYY Identified by HPLC‐MS/MS

**FIGURE 1:**
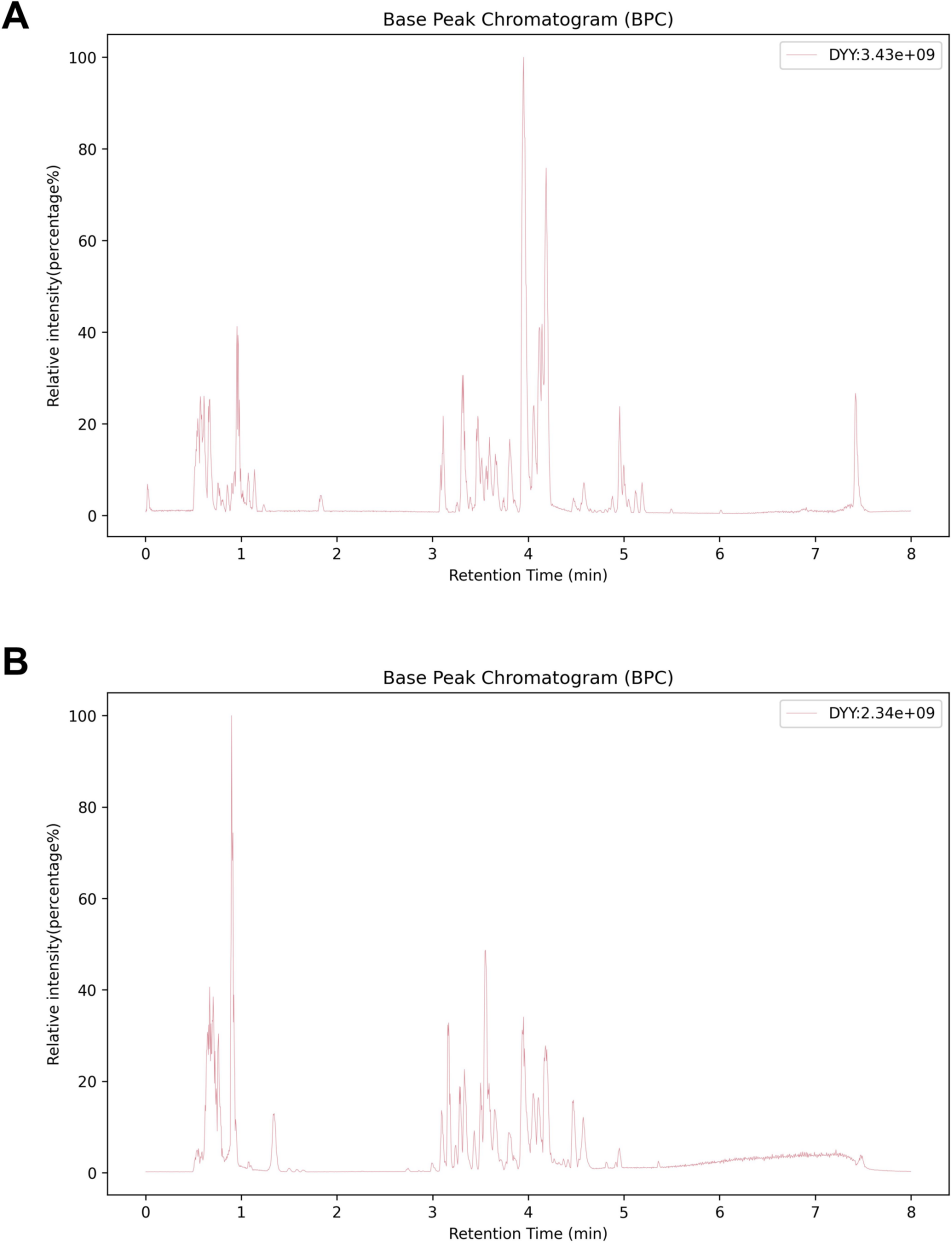
Qualitative analysis of compounds in Da Yuan Yin (DYY) by high‐performance liquid chromatography–tandem mass spectrometry. (A) Total ion peak diagram of DYY in positive ion mode. (B) Total ion peak diagram of DYY in negative ion mode.

The chemical profile of DYY was characterized using HPLC‐MS/MS in both positive and negative ion modes. Representative total ion chromatograms were presented in Figure 1A,B. A total of 40 compounds with high identification scores (Top‐20 in each ion mode) were tentatively identified based on accurate mass, MS/MS fragmentation, and database matching. The detailed list of these compounds, including observed *m*/*z* and putative identities, was summarized in Tables 1 and 2 (positive and negative modes, respectively). This chemical profiling provides a material basis for further pharmacological investigation of DYY in sepsis.

**TABLE 1:** Analysis of active compounds in positive ion mode.

ID	NameEN	Formula	mzmed	rtmed [min]
1	(−)‐Epicatechin	C_15_H_14_O_6_	291.08676	3.303595
2	Methioninesulfoxide	C_5_H_11_NO_3_S	166.0536	0.63507074
3	L‐ARGININE	C_6_H_14_N_4_O_2_	175.1189	0.5791743
4	L‐TYROSINE	C_9_H_11_NO_3_	182.08133	0.9900883
5	Senkyunolide A	C_12_H_16_O_2_	193.12253	5.223154
6	Procyanidin B1	C_30_H_26_O_12_	579.14923	3.1486633
7	Puerarin	C_21_H_20_O_9_	417.11896	3.3556762
8	Isoliquiritigenin	C_15_H_12_O_4_	257.08084	3.6144943
9	LATIFOLICININ C ACID	C_9_H_10_O_4_	165.05493	0.9900883
10	(2E)‐1‐(2,4‐dihydroxyphenyl)‐3‐(4‐hydroxyphenyl)‐2‐propen‐1‐one	C_15_H_12_O_4_	257.08066	3.8625848
11	4‐Hydroxy‐3‐methoxycinnamaldehyde	C_10_H_10_O_3_	179.07062	4.087899
12	1‐methyl‐1,2,3,4‐tetrahydro‐beta‐carboline‐3‐carboxylic acid	C_13_H_14_N_2_O_2_	188.07083	3.0872865
13	N6, N6, N6‐Trimethyl‐L‐lysine	C_9_H_20_N_2_O_2_	189.16017	0.5791743
14	Albiflorin	C_23_H_28_O_11_	481.1715	3.8766067
15	L‐(−)‐Asparagine	C_4_H_8_N_2_O_3_	133.06099	0.6211017
16	Antiarol	C_9_H_12_O_4_	185.08086	3.3363545
17	L‐Threonine	C_4_H_9_NO_3_	120.06549	0.62575907
18	Tetramethylscutellarein	C_19_H_18_O_6_	343.1172	5.0122313
19	Aurantiamide acetate	C_27_H_28_N_2_O_4_	445.21353	5.176329
20	Licochalcone A	C_21_H_22_O_4_	271.09592	4.39427

**TABLE 2:** Analysis of active compounds in negative ion mode.

ID	NameEN	Formula	mzmed	rtmed [min]
1	2‐Isopropylmalic acid	C_7_H_12_O_5_	175.06145	3.2720287
2	Uridine	C_9_H_12_N_2_O_6_	243.06201	0.9316847
3	L‐Histidine	C_6_H_9_N_3_O_2_	154.06206	0.57577187
4	Rhamnetin	C_16_H_12_O_7_	315.05093	4.7111874
5	Tartaric acid	C_4_H_6_O_6_	149.00926	0.66929907
6	Procyanidin B3	C_30_H_26_O_12_	577.1336	3.1303344
7	Desaminotyrosine	C_9_H_10_O_3_	165.05568	3.238935
8	L‐Malic acid	C_4_H_6_O_5_	133.01407	0.76313114
9	D‐Glucuronic acid	C_6_H_10_O_7_	193.0356	0.627385
10	N‐Acetyl‐L‐phenylalanine	C_11_H_13_NO_3_	206.08226	3.6842916
11	Succinis acid	C_4_H_6_O_4_	117.01952	1.0768659
12	Trehalose	C_12_H_22_O_11_	341.10876	0.6599841
13	Citric acid	C_6_H_8_O_7_	191.01979	0.9036409
14	Wogonoside	C_22_H_20_O_11_	459.09244	4.1803727
15	3,4‐Dihydroxy‐5‐methoxybenzoic acid	C_8_H_8_O_5_	183.03026	3.069462
16	Galactarate	C_6_H_10_O_8_	209.03046	0.6320428
17	DL‐N‐Acetyltryptophan	C_13_H_14_N_2_O_3_	203.08295	3.0600972
18	4,4′‐dihydroxy‐2′‐methoxychalcone	C_16_H_14_O_4_	269.0825	4.3862348
19	Atranol	C_8_H_8_O_3_	151.04005	4.4143353
20	Spiculisporic acid	C_17_H_28_O_6_	327.1816	5.2706795

### 3.2 DYY Alleviates Intestinal Injury in Sepsis Mice

**FIGURE 2:**
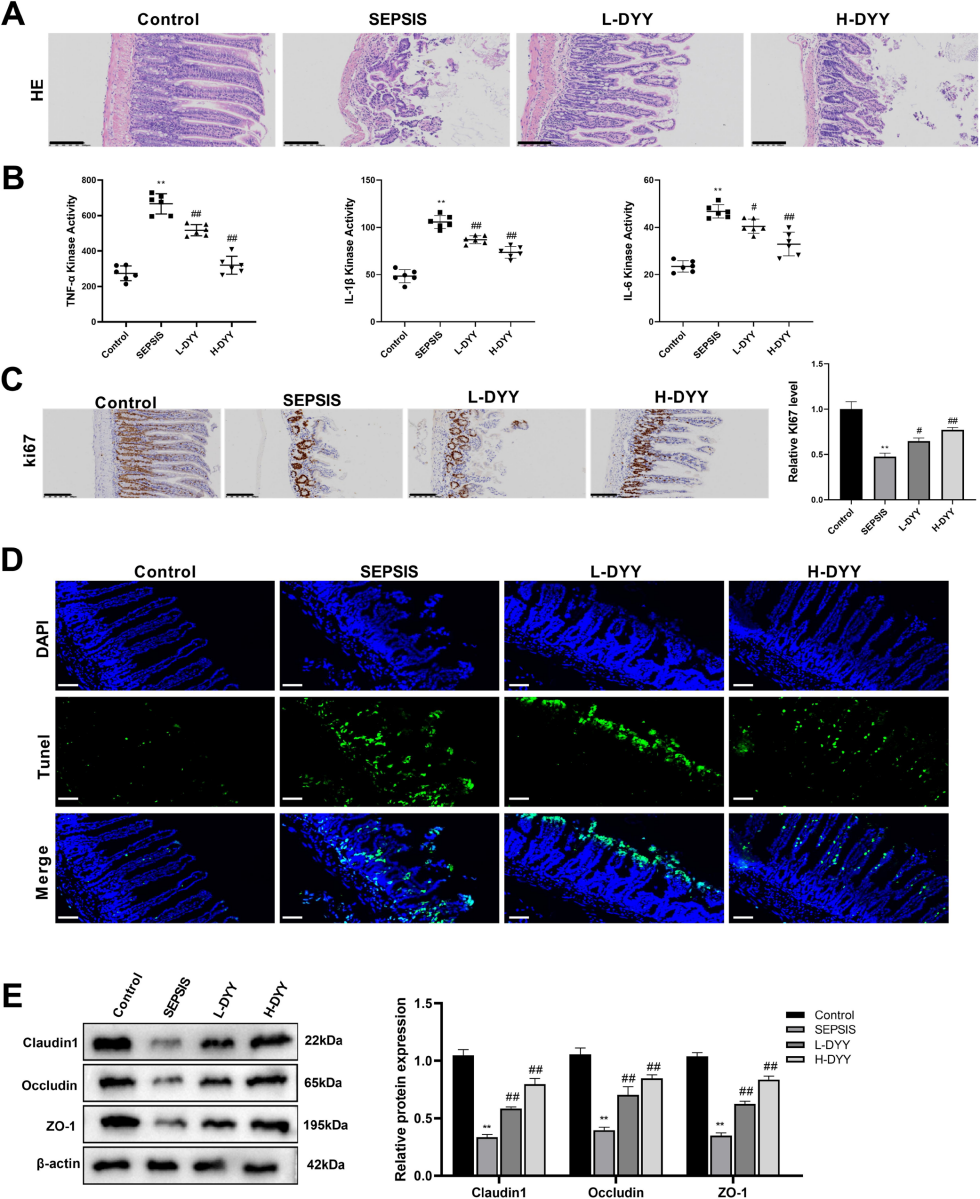
DYY alleviates intestinal injury in sepsis mice. (A) Hematoxylin–eosin (HE) staining was performed to observe histopathologic features of the ileum (magnification: 200×, scale: 100 μm). (B) Enzyme‐linked immunosorbent assay (ELISA) was executed to detect levels of inflammatory factors. (C) Immunohistochemistry (IHC) detection of Ki67 expression (magnification: 200×, scale: 100 μm). (D) Terminal deoxynucleotidyl transferase‐dUTP nick‐end labeling (TUNEL) staining was performed to detect apoptosis. (E) Western blot was carried out to detect protein expression of Claudin‐1, Occludin, and ZO‐1. **p < 0.01 versus Control; ^#^ *p* < 0.05 versus SEPSIS; ^##^ *p* < 0.01 versus SEPSIS.

HE staining was used to observe the ileal tissue morphology of mice (Figure 2A), which showed that in the Control group, the intestinal villi of mice were clearly arranged, with the regular structure and the complete lamina propria. In the SEPSIS group, the intestinal mucosa became thinner and lost integrity, and the gap with the lamina propria increased, with disorder arrangement and inflammatory cell infiltration. Compared with the SEPSIS group, L‐DYY slightly narrowed the gap between the intestinal mucosa and lamina propria in mice, with a slightly more regular arrangement of villi; H‐DYY significantly increased intestinal mucosal thickness, with significantly better villus length and alignment and intact lamina propria (Figure 2A).

ELISA results demonstrated that compared with the Control group, the concentrations of TNF‐α, IL‐1β, and IL‐6 in the SEPSIS group were significantly increased. And compared with the SEPSIS group, L‐DYY and H‐DYY significantly reduced the levels of TNF‐α, IL‐1β, and IL‐6 (*p* < 0.01) (Figure 2B).

Furthermore, IHC was carried out to measure cell proliferation, demonstrating that compared with the Control group, mice in the SEPSIS group showed a significant decrease in Ki67 positive cells in the ileocecal villi, and L‐DYY and H‐DYY increased Ki67 positive cells in the ileocecal villi compared with the SEPSIS group (Figure 2C). Apoptosis was detected by TUNEL staining, showing that compared with the Control group, the level of apoptosis in the ileal tissues was significantly higher in the SEPSIS group; L‐DYY and H‐DYY significantly suppressed apoptosis compared with the SEPSIS group (Figure 2D).

The expression levels of tight junction‐related proteins in the ileum tissues were detected by western blot, showing that compared with the Control group, the protein expression levels of Claudin‐1, Occludin, and ZO‐1 in the SEPSIS group were significantly reduced (*p* < 0.01) (Figure 2E). Compared with the SEPSIS group, the expression levels of Claudin‐1, Occludin, and ZO‐1 protein in the L‐DYY group and H‐DYY group were significantly raised (*p* < 0.01) (Figure 2E).

### 3.3 Intestinal Species Composition Analysis

**FIGURE 3:**
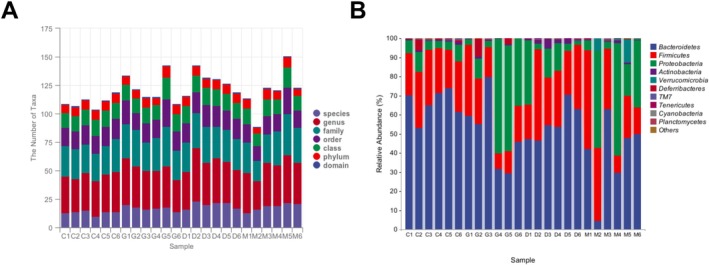
Intestinal microbial community composition across different groups. (A) Accumulation of the number of microbial taxonomic units at different taxonomic levels (from domain to species) in individual samples. The stacked bars represent the abundance of taxa from domain (bottom) to species (top) for each sample (C1–C6,G1–G6, D1–D6, M1–M6). (B) Relative abundance distribution of intestinal microbial communities at the phylum level. The histogram displays the proportional composition of major bacterial phyla across all samples. (C: control, M: model, G: high‐DYY, D: low‐DYY).

The intestinal contents in the four groups of mice were collected for 16S rRNA sequencing. A total of 1,548,054 high‐quality sequences were acquired from the samples, with an average length of 422.08 base pairs. The statistical diagram of the number of microbial taxonomic units showed the number of taxonomic units contained in different samples at the seven levels of domain, phylum, class, order, family, genus, and species (Figure 3A). The composition distribution of each sample at the family and genus levels was displayed in Figure 3B.

### 3.4 Intestinal Species Diversity Analysis

**FIGURE 4:**
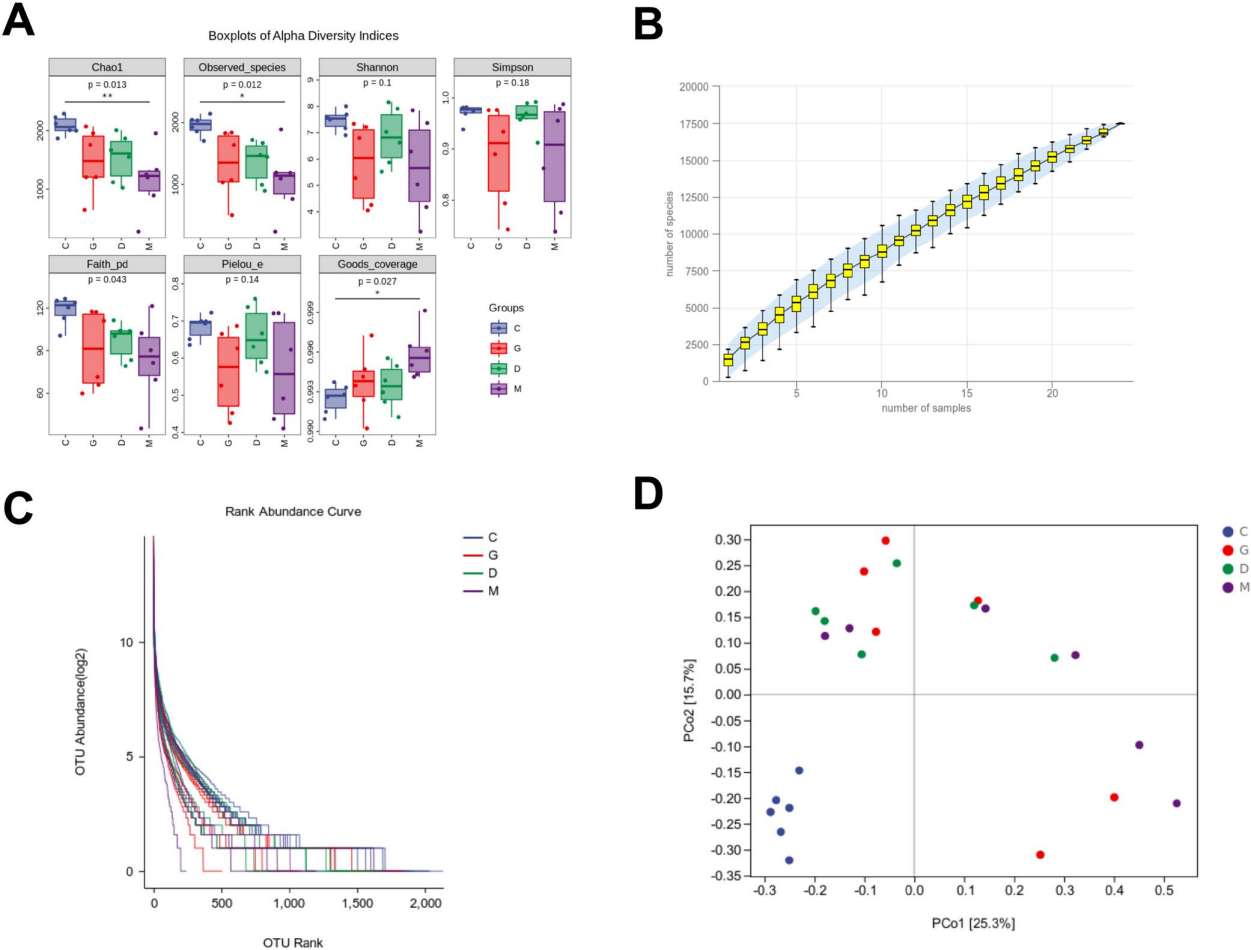
Species diversity analysis. (A) Boxplots of Alpha diversity index. (B) Specaccum species accumulation plot. (C) Rank abundance curve. (D) 2D plot of PCoA analysis (C: Control, D: Low‐DYY, G: High‐DYY, M: Model).

Alpha diversity analysis (Figure 4A) showed that based on Chao1 and observed‐species indexes, the microbial community diversity in the SEPSIS group was significantly lower than that in the Control group. Compared with the SEPSIS group, the microbial community diversity in the L‐DYY group and the H‐DYY group was significantly increased, and the microbial community diversity in the H‐DYY group was slightly higher than that in the L‐DYY group. Based on the Goods‐coverage index, the microbial community diversity in the SEPSIS group was significantly higher than that in the Control group. Compared with the SEPSIS group, the microbial community diversity in the L‐DYY group and the H‐DYY group was significantly reduced. Other indexes (Pielou's evenness, Faith's PD, Simpson, Shannon) indicated that there was no significant difference between the four groups (Figure 4A).

The species accumulation curve showed that the curve tended to be gentle with the addition of new samples, indicating that the sample size was sufficient to reflect the species composition of the community (Figure 4B). In addition, the gentleness of the abundance grade curve reflected the uniformity of microbial composition (Figure 4C).

PCoA analysis showed that in the first principal component PC1 (difference ratio 25.3%) and the second principal component (difference ratio 15.7%) coordinate axes, the Control group and the SEPSIS group were on the opposite side and far away, and the SEPSIS group was on the opposite side and far away from the L‐DYY group and the H‐DYY group (Figure 4D). It was proved that there were differences in intestinal flora between the Control group and the SEPSIS group, and DYY treatment could change the intestinal flora structure of the SEPSIS group.

### 3.5 Gut Microbiota Analysis

**FIGURE 5:**
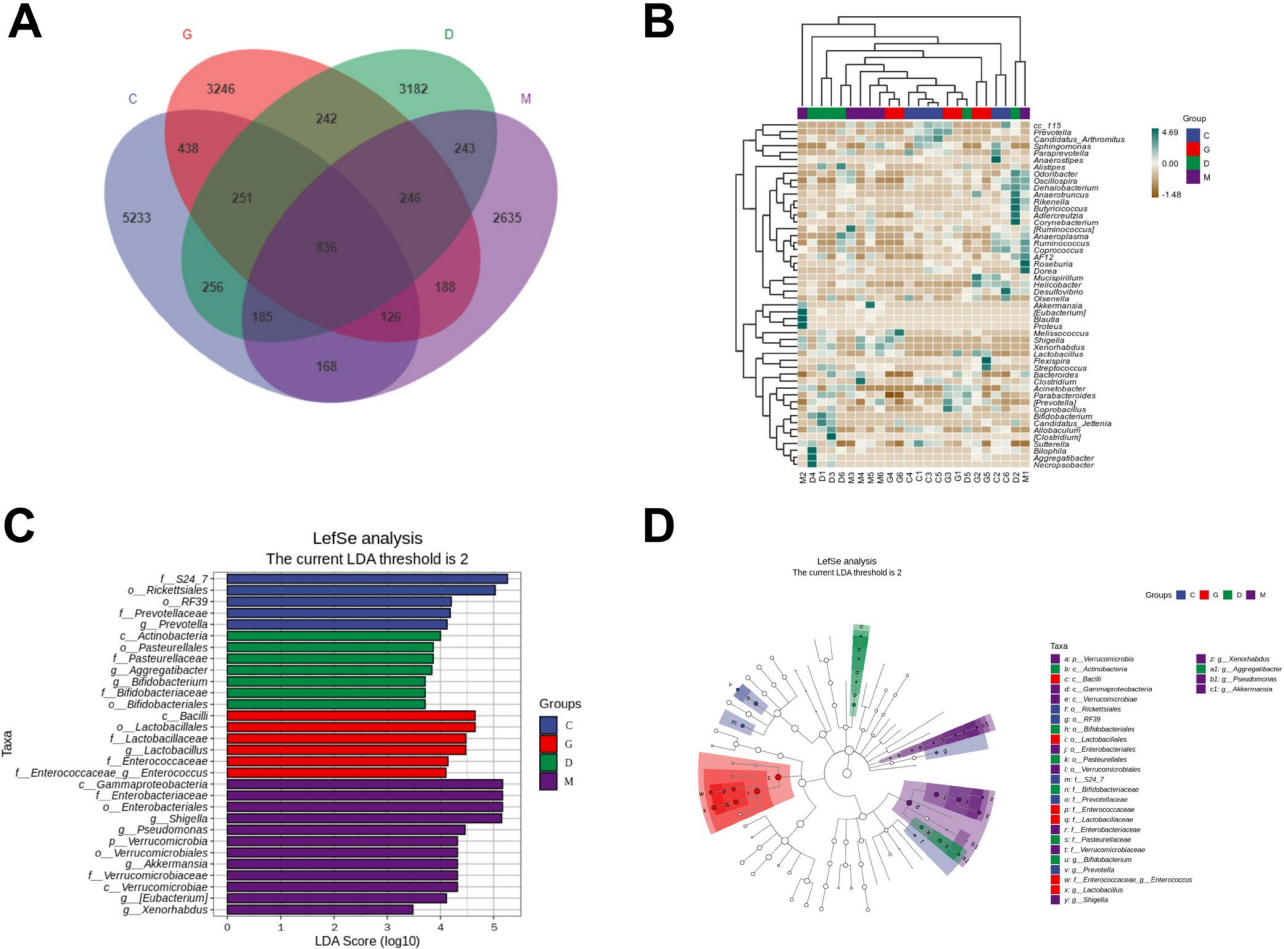
Analysis of species differences. (A) Venn diagram. (B) Heat map of species composition at genus level. (C) Histogram of Linear discriminant analysis Effect Size (LefSE) analysis for marker species. (D) Taxonomic branching map of LefSE analysis (C: Control, D: Low‐DYY, G: High‐DYY, M: Model).

The Venn diagram showed that in the Control group, SEPSIS group, L‐DYY group, and H‐DYY group, 5233, 2635, 3182, and 3246 ASVs were detected separately, and 836 common ASVs were detected (Figure 5A).

To further compare the species composition of the samples, the top 50 genera with average abundance were used to draw the heat map to show the species abundance distribution trend of each sample (Figure 5B). In addition, the histogram of the LDA value distribution in the top five groups with significant differences was obtained by the LEfSe analysis method to show the species significantly enriched in each group and their importance, demonstrating that the intestinal microbiome in sepsis mice was dominated by GammaProteobacteria, Enterobacteriaceae, Shigella, Pseudomonas, Verrucomicrobia, and Akkermansia, and H‐DYY reduced this alteration and promoted the growth of Bacilli in the intestine, such as Lactobacillales and Enterobacteriaceae (Figure 5C). The species taxonomy branch diagram showed the taxonomic hierarchy distribution of the markers in each group (Figure 5D).

### 3.6 Effect of DYY on the Metabolic Function of Gut Microbiota in Sepsis Mice

The metabolic function of gut microbiota in mice was predicted by PICRUst analysis. The results showed that compared with the Control group, there were 31 functional metabolic pathways affected by the gut microbiota in the SEPSIS group. Among them, the functional pathways with increased abundance in the L‐DYY and H‐DYY groups included amino acid metabolism, lipopolysaccharide biosynthesis, peptidoglycan synthesis, pantothenic acid, and coenzyme a biosynthesis, secondary bile acid biosynthesis, and so forth (Table 3).

**TABLE 3:** Metabolic function of gut microbiota predicted by PICRUst analysis.

Pathway	Description	C (Control)	G (High DYY)	D (Low DYY)	M (Model)
ko00290	Valine, leucine and isoleucine biosynthesis	850.5198333	678.7511667	763.2535	729.0368333
ko00471	D‐Glutamine and D‐glutamate metabolism	845.3005	778.638	813.4343333	696.5105
ko00250	Alanine, aspartate and glutamate metabolism	769.9128333	630.6066667	713.6848333	641.2911667
ko00670	One carbon pool by folate	725.3248333	641.1063333	704.932	610.937
ko00550	Peptidoglycan biosynthesis	709.086	653.5535	690.9436667	620.1981667
ko00121	Secondary bile acid biosynthesis	695.5913333	531.8155	597.928	411.6188
ko00770	Pantothenate and CoA biosynthesis	689.9148333	601.3088333	632.6876667	590.6055
ko00710	Carbon fixation in photosynthetic organisms	663.5155	574.4778333	641.1655	582.5678333
ko00780	Biotin metabolism	642.306	589.5726667	564.3548333	543.315
ko03430	Mismatch repair	638.7358333	587.6773333	623.342	573.2531667
ko00970	Aminoacyl‐tRNA biosynthesis	631.4365	586.0361667	603.7536667	529.7763333
ko00300	Lysine biosynthesis	626.722	546.1255	594.175	533.641
ko03010	Ribosome	625.879	574.2893333	601.4508333	500.7955
ko04112	Cell cycle—Caulobacter	594.4973333	543.8233333	578.4663333	521.0746667
ko03440	Homologous recombination	594.0606667	544.073	587.4101667	523.0885
ko03060	Protein export	568.955	540.2705	549.1981667	498.1035
ko00061	Fatty acid biosynthesis	567.2543333	564.915	540.804	508.2036667
ko00790	Folate biosynthesis	510.2305	500.907	520.9568333	469.0528333
ko00900	Terpenoid backbone biosynthesis	505.4715	450.6003333	481.2243333	418.9233333
ko00400	Phenylalanine, tyrosine and tryptophan biosynthesis	490.0691667	393.5141667	444.252	407.3018333
ko03030	DNA replication	478.2136667	446.8996667	472.6783333	420.3866667
ko00720	Carbon fixation pathways in prokaryotes	461.976	423.3708333	430.9375	395.6725
ko00750	Vitamin B6 metabolism	452.9245	445.3215	455.0723333	405.4643333
ko00240	Pyrimidine metabolism	449.4478333	425.6885	442.575	396.6163333
ko00540	Lipopolysaccharide biosynthesis	440.5811667	464.9528333	406.8745	348.5458333
ko00760	Nicotinate and nicotinamide metabolism	425.1391667	407.6528333	421.8158333	377.768
ko00020	Citrate cycle (TCA cycle)	420.5561667	407.1845	401.7855	376.5858333
ko00051	Fructose and mannose metabolism	397.558	419.1016667	423.403	449.1966667
ko00531	Glycosaminoglycan degradation	355.5376667	268.1633333	338.7261667	257.8206
ko00908	Zeatin biosynthesis	311.1708333	271.2318333	294.9371667	237.0818333
ko00740	Riboflavin metabolism	307.6711667	293.3483333	278.1073333	271.3348333

**FIGURE 6:**
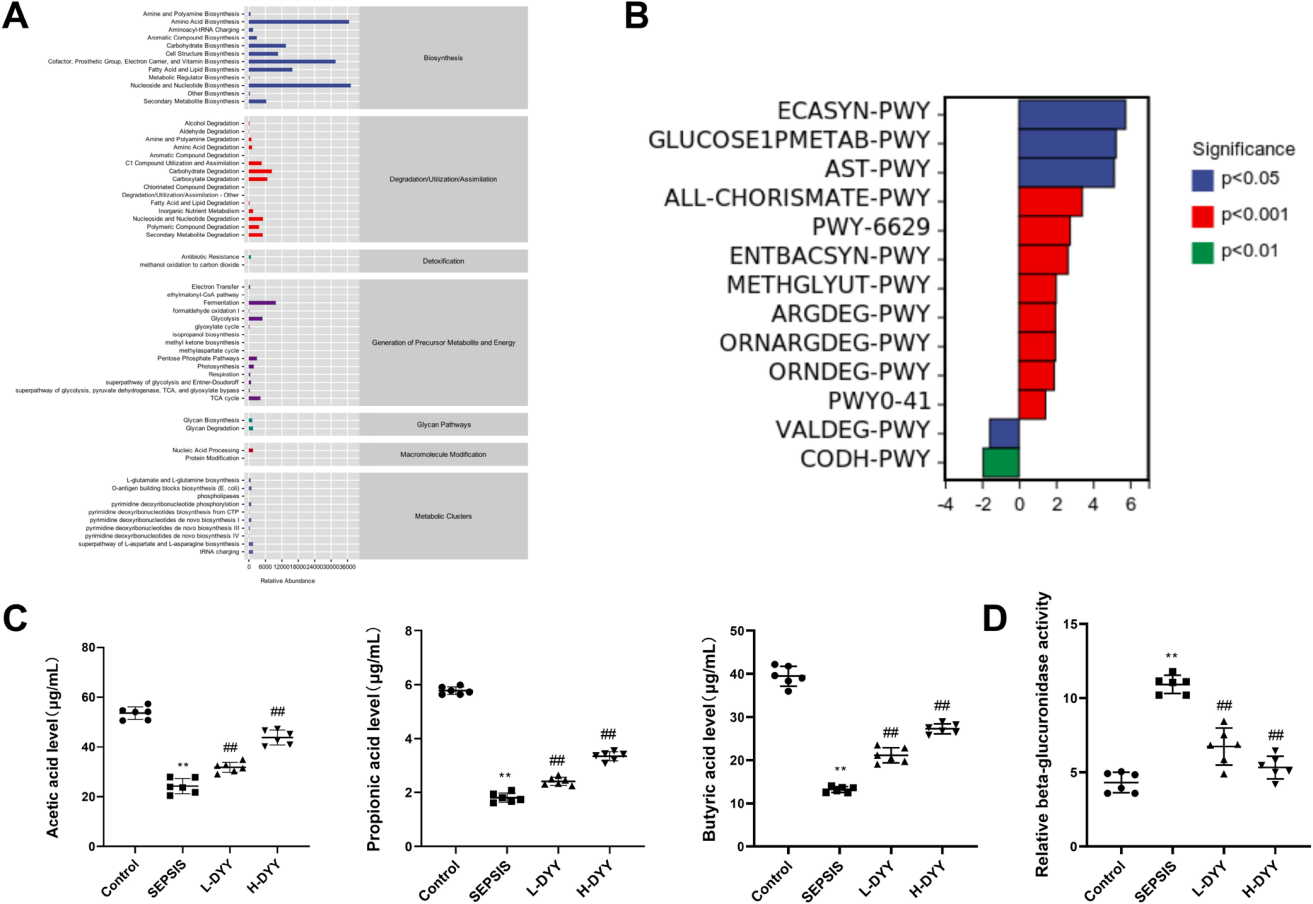
Effect of DYY on the metabolic function of gut microbiota in Sepsis mice. (A) Abundance map of KEGG secondary functional pathway. (B) KEGG metabolic pathways differing between Control and SEPSIS groups. (C) Concentrations of short‐chain fatty acids (acetate, propionate, butyrate) in intestinal contents were measured. (D) β‐Glucuronidase activity in intestinal contents was measured. *p < 0.05, **p < 0.01 versus Control group; ^#^ *p* < 0.05, ^##^ *p* < 0.01 versus SEPSIS group.

The abundance of secondary functional pathways based on the KEGG database was counted, and a total of 58 pathways were altered (Figure 6A). The KEGG metabolic pathways with differences between groups were obtained using metagenomeSeq, demonstrating that compared with the Control group, the SEPSIS group increased the abundance of pathways such as biosynthesis of common antigens of Enterobacteriaceae, glucose degradation, AST pathway, chorismate metabolic pathway, and tryptophan biosynthesis (Figure 6B). There was no significant change in the pathway abundance values in the L‐DYY and H‐DYY groups compared to the SEPSIS group.

To experimentally assess functional changes in the gut microbiota beyond genomic predictions, we quantified the levels of key microbial metabolites and enzymatic activity. Consistent with a state of dysbiosis and impaired microbial fermentation, the concentrations of beneficial SCFAs—acetate, propionate, and butyrate—were significantly decreased in the intestinal contents of septic mice compared to the Control group (Figure 6C). DYY treatment, particularly at the H‐DYY, markedly reversed this deficit, leading to a significant increase in all three SCFAs compared to the SEPSIS group (Figure 6C). Conversely, the activity of β‐glucuronidase, a bacterial enzyme often associated with the reactivation of toxins and harmful metabolites, was significantly elevated in the SEPSIS group. Treatment with both L‐DYY and H‐DYY effectively suppressed this elevated enzymatic activity (Figure 6D). These findings provide direct experimental confirmation that DYY not only alters the taxonomic profile but also rectifies critical functional outputs of the gut microbiota in sepsis, promoting a metabolic environment characterized by increased production of protective SCFAs and reduced potential for harmful bacterial metabolism.

### 3.7 FMT From DYY‐Treated Donors Alleviates Sepsis‐Induced Intestinal Injury

**FIGURE 7:**
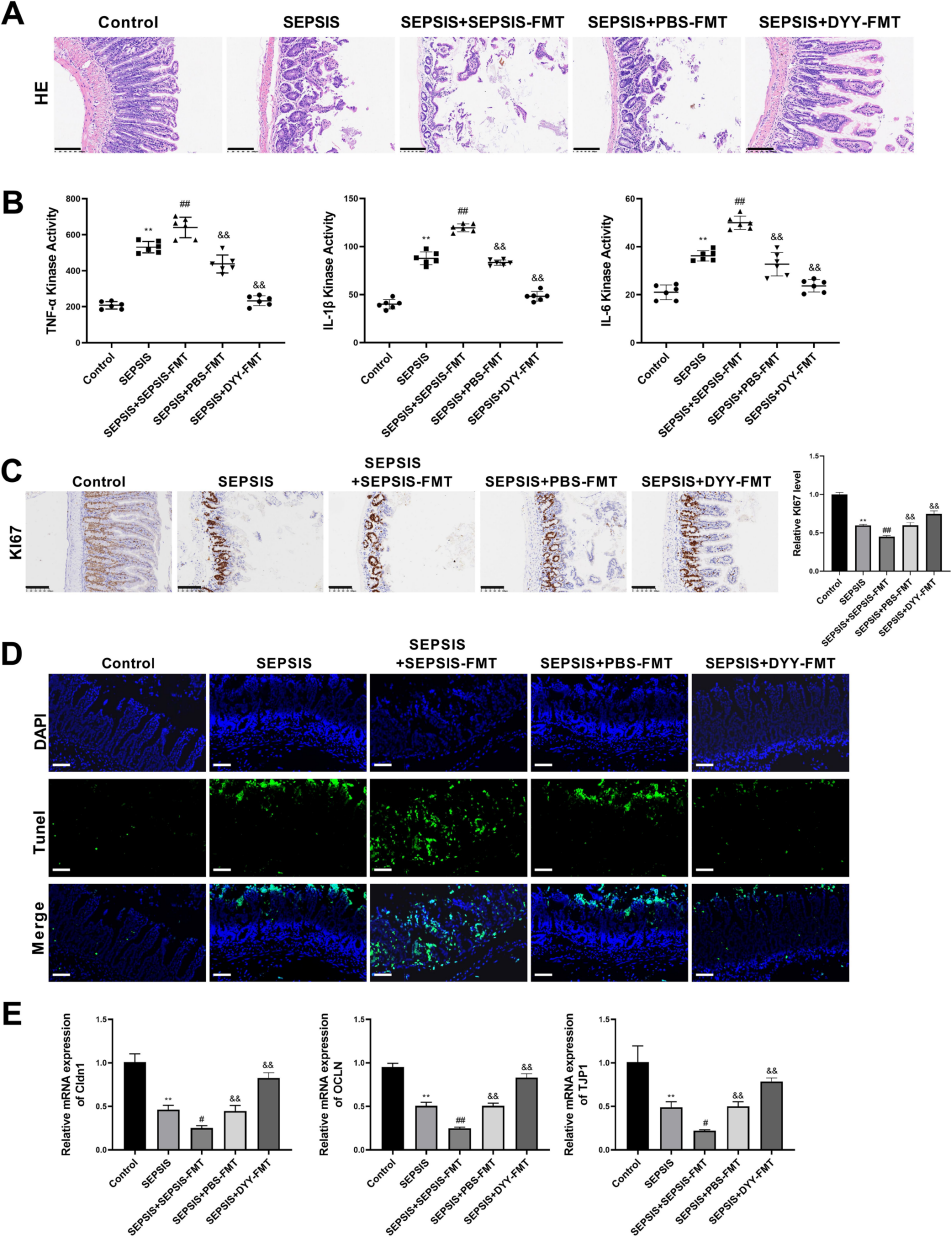
Fecal microbiota transplantation (FMT) from DYY‐treated donors alleviates sepsis‐induced intestinal injury. (A) Representative HE staining images of ileal tissues from different FMT recipient groups (magnification: 200×, scale: 100 μm). (B) Serum levels of TNF‐α, IL‐1β, and IL‐6 measured by ELISA. (C) Representative IHC images (magnification: 200×, scale: 100 μm) and quantification of Ki67‐positive cells. (D) TUNEL staining was performed to detect apoptosis. (E) mRNA expression levels of tight junction genes in ileal tissues measured by qRT‐PCR. **p < 0.01 versus Control group; ^#^ *p* < 0.05; ^##^ *p* < 0.01 versus SEPSIS group; ^&&^ *p* < 0.01 versus SEPSIS + SEPSIS − FMT group.

To determine whether the modulation of gut microbiota was essential for DYY's therapeutic effect, we performed FMT experiments. As shown in Figure 7A, septic recipient mice that received microbiota from septic donors (SEPSIS + SEPSIS − FMT) exhibited severe intestinal mucosal damage, characterized by thinner mucosa, disrupted villi structure, and significant inflammatory infiltration, which was more pronounced than in the SEPSIS group without FMT. In contrast, septic recipients transplanted with microbiota from healthy donors (SEPSIS + PBS − FMT) or from DYY‐treated septic donors (SEPSIS + DYY − FMT) showed markedly ameliorated intestinal pathology, including restored mucosal thickness, orderly villi arrangement, and reduced inflammatory cell infiltration.

Consistently, ELISA results revealed that serum levels of TNF‐α, IL‐1β, and IL‐6 were significantly elevated in the SEPSIS + SEPSIS − FMT group compared to the SEPSIS group. However, these pro‐inflammatory cytokines were significantly reduced in both the SEPSIS + PBS − FMT and SEPSIS + DYY − FMT groups (Figure 7B).

Assessment of proliferation and apoptosis in ileal tissues demonstrated that the reduction in Ki67‐positive cells and the increase in TUNEL‐positive cells observed in the SEPSIS + SEPSIS − FMT group were notably reversed in recipients of healthy or DYY‐modulated microbiota (Figure 7C,D).

Furthermore, qRT‐PCR analysis of tight junction genes showed that the downregulation of *CLDN1* (Claudin‐1), *OCLN* (Occludin), and *TJP1* (ZO‐1) in the SEPSIS + SEPSIS − FMT group was significantly attenuated in the SEPSIS + PBS − FMT and SEPSIS + DYY − FMT groups (Figure 7E). Collectively, these data demonstrate that the protective effects of DYY against intestinal injury can be transferred via FMT, providing direct evidence that the regulation of gut microbiota is a key mechanism through which DYY exerts its therapeutic benefits in sepsis.

## 4 Discussion

Sepsis, a life‐threatening organ dysfunction caused by a dysregulated host response to infection, usually causes significant morbidity and mortality (Zhang and Ning 2021). The intricate pathophysiology of sepsis involves a complex interaction between the immune system, inflammatory responses, and the gut microbiota. Recently, the gut microbiota has emerged as a critical player in sepsis pathogenesis, with alterations in its composition and function contributing to intestinal injury and systemic inflammation (Yang et al. 2024). In our study, in vivo experiments revealed that DYY inhibited intestinal inflammation and apoptosis and promoted the levels of tight junction proteins to ameliorate intestinal injury. Through 16S rRNA sequencing and metabolome sequencing, it was found that DYY regulated the intestinal microbiota in sepsis mice and promoted the growth of Bacilli, such as Lactobacillales and Enterobacteriaceae.

The intestine is a highly complex system in which the immune system, intestinal epithelial cells, and microbiota maintain balance in various ways (Sardinha‐Silva et al. 2022). The damage of the intestinal mucosal barrier may lead to bacterial translocation, causing infection and resulting in multiple organ failure. In sepsis patients, the intestine can provide a place for pathogen growth, which leads to intestinal damage, destroys intestinal barrier protection, and further aggravates sepsis (Liu et al. 2023). Tight junction proteins are of vital significance in maintaining intestinal barrier function. Tight junctions are formed by a variety of adhesion proteins, including occludin, claudins, and zonula occludens (ZO) family proteins (Kuo et al. 2022). It is reported that plasma levels of tight junction‐related proteins (occludin and ZO‐1) are positively correlated with the severity of sepsis (Zhao et al. 2016). In sepsis, Tongfufei decoction inhibits inflammation and raises the expression of ZO‐1/Occludin/Claudin‐1 to alleviate intestinal mucosal injury and maintain intestinal barrier function (Chen et al. 2020). In our study, the intestinal mucosa became thinner and the expressions of intestinal tight junction proteins (Claudin‐1, ZO‐1, and Occludin) were decreased in sepsis mice, while DYY significantly improved this phenomenon and promoted the expression of tight junction proteins, which was similar to previous studies. In Wang's research, rhubarb monomers enhance the levels of junction proteins to protect the intestinal barrier and ameliorate mucosal damage in sepsis (Wang et al. 2017). Shenfu decoction improves intestinal permeability by promoting the levels of ZO‐1, Occludin, Claudin‐1, and p‐VASP, thereby alleviating intestinal damage and inflammation caused by sepsis (Liu et al. 2021).

Gut microbiota is strongly associated with the progression of sepsis (Zhou et al. 2023). In sepsis, the composition of the intestinal microbial flora is destroyed, resulting in the death of symbiotic bacteria and the excessive reproduction of other pathogens (Kullberg et al. 2021). Studies have reported that fecal microbiota transplantation and short‐chain fatty acids play a protective role in sepsis, which can increase Occludin expression, inhibit inflammation and pyroptosis, and regulate intestinal bacterial abundance to reduce sepsis mortality (Lou et al. 2022). Furthermore, many Chinese herbal medicines and their extractions have been proved to alleviate the progression of sepsis by regulating the microbiota. Nobiletin, a plant‐derived polymethoxyflavone, can prevent ferroptosis and activate the NRF signaling pathway by regulating the gut microbiota to reduce sepsis‐related acute liver injury (Huang et al. 2023). Jinhong decoction reduces intestinal bacterial translocation and improves intestinal microbial homeostasis to protect sepsis‐related ALI (Bao et al. 2023). Mu et al. report that Xuanbai Qingqi decoction is able to regulate the intestinal microbiota, restore the intestinal epithelial barrier, and inhibit inflammation response to alleviate the progression of sepsis (Mu et al. 2021). Sini decoction improves lung injury induced by cecal ligation and puncture in mice by regulating the composition of intestinal microflora (Wang et al. 2020). In this paper, through 16S rRNA sequencing analysis, it was found that DYY could regulate the gut microbiota of septic mice, further confirming the role of DYY in alleviating sepsis. Our FMT experiment provides direct functional evidence that the gut microbiota plays a causal role in DYY‐mediated protection. Transferring microbiota from DYY‐treated septic donors reproduced the therapeutic benefits—reduced inflammation, improved epithelial integrity, and enhanced tight‐junction expression—in recipient septic mice. This confirms that DYY's protective effects are indeed mediated through its modulation of the gut‐microbial community.

In summary, in vivo assay demonstrated that DYY can inhibit inflammation, apoptosis, and promote levels of tight junction related proteins to alleviate intestinal injury in sepsis. Moreover, through 16S rRNA sequencing and metabolome sequencing, it was manifested that DYY regulated gut microflora in sepsis. While our PICRUSt2‐based analysis did not detect significant overall shifts in predicted metabolic pathway abundance, this does not preclude the possibility that DYY may influence specific metabolic functions that are below the detection limit of the current methodology. Our direct measurements of SCFAs and β‐glucuronidase activity confirmed that DYY meaningfully influenced microbial metabolism. The FMT experiment definitively established that these benefits were mediated through gut microbiota remodeling. Our study provides evidence that DYY regulates the gut microbiota and improves intestinal injury in sepsis. This TCM formula offers a prospective therapeutic approach for the management of sepsis, particularly in light of the increasing recognition of the gut microbiota as a critical modulator of sepsis outcomes.

## Author Contributions

**Jian Guo:** supervision, project administration, writing – review and editing.

## Funding

This work was supported by Natural Science Foundation of Shanghai (23ZR1464200).

## Ethics Statement

The animal experiments were approved by the local ethical committee of Yueyang Hospital of Integrated Traditional Chinese and Western Medicine, Shanghai University of Traditional Chinese Medicine (YYLAC‐2022‐W) in accordance with the ARRIVE guidelines.

## Conflicts of Interest

The authors declare no conflicts of interest.

## Supporting information


**Table S1:** fsn371456‐sup‐0001‐TableS1.docx.

**Table S2:** fsn371456‐sup‐0002‐TableS2.xlsx.

## Data Availability

The data and materials supporting the findings of this study are available from the corresponding authors upon request.
