# Supplementary material for: Da Yuan Yin Regulates Gut Microbiota and Improves Intestinal Injury in Sepsis
Source: Food Sci Nutr. 2026 Apr 3;14(4):e71456. doi: 10.1002/fsn3.71456 (PMC13051883; doi:10.1002/fsn3.71456)
Supplement: Supplementary file 1 — Table S1: fsn371456‐sup‐0001‐TableS1.docx. [file FSN3-14-e71456-s002.docx]

**Table S1 Primer sequences for RT-PCR in this study**

| Primer name | Sequences (5’-3’) |
| --- | --- |
| GAPDH-F | GAGTCAACGGATTTGGTCGT |
| GAPDH-R | TTGATTTTGGAGGGATCTCG |
| CLDN1-F | TGGGGCTGATCGCAATCTTT |
| CLDN1-R | CAGAGGGAAGCAGCAGTTCA |
| OCLN-F | TTTCCTGCGGTGACTTCTCC |
| OCLN-R | AAGGACTGGGCAATCACCTG |
| TJP1-F | TCCTGACCAACGTTCAGAGC |
| TJP1-R | AGGACGGCCTCTTCCCTTAT |
